# Single-Dose Intranasal Treatment with DEF201 (Adenovirus Vectored Consensus Interferon) Prevents Lethal Disease Due to Rift Valley Fever Virus Challenge

**DOI:** 10.3390/v6031410

**Published:** 2014-03-24

**Authors:** Brian B. Gowen, Jane Ennis, Kevin W. Bailey, Zachary Vest, Dionna Scharton, Eric J. Sefing, Jeffrey D. Turner

**Affiliations:** 1 Department of Animal, Dairy and Veterinary Sciences, Utah State University, Logan, UT 84322, USA; E-Mails: kevin.bailey@aggiemail.usu.edu (K.W.B.); zach.v@aggiemail.usu.edu (Z.V.); dionna.scharton@aggiemail.usu.edu (D.S.); biosefing@gmail.com (E.J.S.); 2 Institute for Antiviral Research, Utah State University, Logan, UT 84322, USA; 3 School of Veterinary Medicine, Utah State University, Logan, UT 84322, USA; 4 Defyrus Inc., 2 Bloor Street W, Suite 2602, Toronto, Ontario, M4W 3E2, Canada; E-Mails: jane.ennis@defyrus.com (J.E.); jeff.turner@defyrus.com (J.D.T.)

**Keywords:** Rift Valley Fever Virus (RVFV), phlebovirus, bunyavirus, interferon alpha, DEF201, antiviral

## Abstract

Rift Valley fever virus (RVFV) causes severe disease in humans and ungulates. The virus can be transmitted by mosquitoes, direct contact with infected tissues or fluids, or aerosol, making it a significant biological threat for which there is no approved vaccine or therapeutic. Herein we describe the evaluation of DEF201, an adenovirus-vectored interferon alpha which addresses the limitations of recombinant interferon alpha protein (cost, short half-life), as a pre- and post-exposure treatment in a lethal hamster RVFV challenge model. DEF201 was delivered intranasally to stimulate mucosal immunity and effectively bypass any pre-existing immunity to the vector. Complete protection against RVFV infection was observed from a single dose of DEF201 administered one or seven days prior to challenge while all control animals succumbed within three days of infection. Efficacy of treatment administered two weeks prior to challenge was limited. Post‑exposure, DEF201 was able to confer significant protection when dosed at 30 min or 6 h, but not at 24 h post-RVFV challenge. Protection was associated with reductions in serum and tissue viral loads. Our findings suggest that DEF201 may be a useful countermeasure against RVFV infection and further demonstrates its broad-spectrum capacity to stimulate single dose protective immunity.

## 1. Introduction

Rift Valley fever virus (RVFV; *Bunyaviridae*, *Phlebovirus*) has been the cause of multiple epizootics throughout sub-Saharan Africa and the Arabian Peninsula [1]. It is a mosquito-borne virus that causes significant losses in livestock largely from high mortality in newborn animals [2]. RVFV transmission to humans occurs through the bite of infected mosquitoes or contact with tissue from infected animals. The infection generally causes a mild febrile illness, but can lead to severe disease in the form of retinitis, fulminant hepatitis, encephalitis, or viral hemorrhagic fever, with the mortality rate estimated at 10%–20% for hospitalized patients [3,4]. Presently, there are no FDA-approved vaccines or antivirals to prevent or treat RVFV infection, which highlights the need to develop new intervention strategies. Because the virus is also infectious by the airborne route, it poses a potential bioterrorism threat, which is amplified by the fact that mosquitoes native to the United States can readily transmit RVFV and serve as vectors [5]. To this end, the Centers for Disease Control and Prevention (CDC) and the National Institute of Allergy and Infectious Diseases (NIAID) have listed RVFV as a priority Category A pathogen [6]. In addition, RVFV is classified as an overlap select agent regulated by the US Departments of Health and Human Services (DHHS) and Agriculture (USDA).

DEF201 is an adenovirus-vectored human interferon (IFN) alpha with long-lasting antiviral activity in several *in vivo* models of viral infection [7,8,9], including Punta Toro virus (PTV) [10], a related bunyavirus used to model RVFV infection [11,12]. RVFV is reportedly sensitive to the effects of exogenous interferon (IFN). Previous work modeling RVF disease in macaques demonstrated a correlation between reduced disease severity and early presence of IFN [13]. Moreover, administration of recombinant or native human IFN-alpha were highly effective when given prophylactically 1 day prior or 6 h post RVFV challenge [14]. Despite this success, a number of factors including cost, the requirement for multiple injections by healthcare workers, and high bolus dosing to counter the short half-life have precluded recombinant IFN use. In contrast, DEF201 can be manufactured in large scale at manageable cost using widely available methodology, and using the adenovirus to deliver constitutive *in situ* IFN internally allows for more consistent dosing, thereby reducing IFN-related toxicity associated with bolus dosing [15]. Although the usage of PEGylation has improved the efficacy of IFN and reduced the frequency of dosing, therapy remains costly and invasive [16,17]. Because the dosing route of DEF201 is intranasal, it can be administered by a non-healthcare professional using a simple device, which is essential in an outbreak scenario.

In the present study we evaluated DEF201 as a pre- and post-exposure prophylactic intervention in the hamster RVFV infection model to demonstrate its activity against the highly pathogenic ZH501 strain of the virus. Because RVFV is rapidly lethal in hamsters, post-exposure treatments were administered shortly after challenge and a higher dose of 10^8^ plaque-forming units (PFU) of DEF201 was used. This dose is well tolerated in hamsters and induces considerable levels of IFN for enhancing antiviral activity [10].

## 2. Results

**Figure 1:**
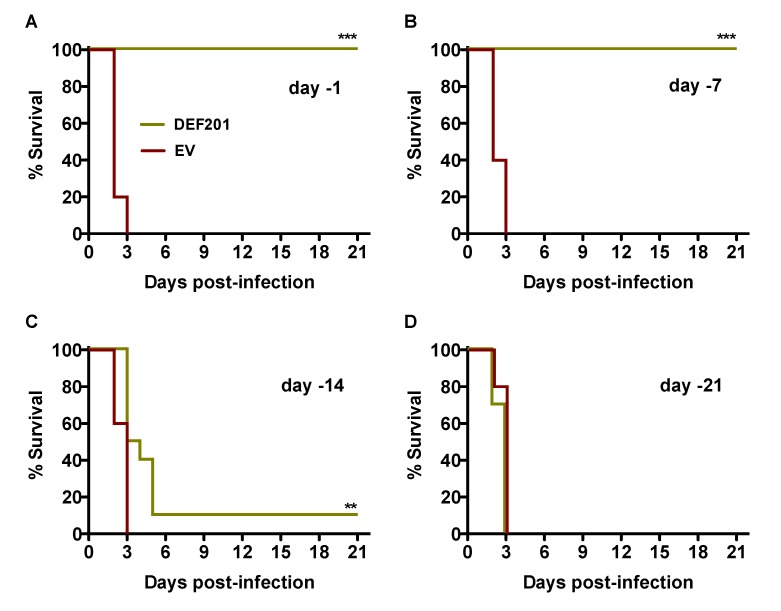
Pre-exposure prophylaxis with DEF201 protects hamsters from lethal s.c. RVFV challenge. Hamsters in each experimental group (n = 10) were treated i.n. with 10^8^ PFU of DEF201 or empty vector (EV) on (**A**) day −1, (**B**) day −7, (**C**) day −14, or (**D**) day −21 relative to the time of infection with 30 PFU of RVFV. Kaplan-Meier survival curves are shown. **p < 0.01, ***p < 0.001 compared to respective EV controls.

We first evaluated DEF201 as a pre-exposure prophylaxis in the hamster RVFV infection model by intranasal (i.n.) instillation at either −21, −14, −7, or −1 day relative to time of subcutaneous (s.c.) challenge. As shown in Figure 1A and B, pre-exposure treatment with 10^8^ PFU of DEF201 at either 1 or 7 days prior to infection provided complete protection from mortality when compared to their respective empty vector (EV)‑treated groups (*p* < 0.001), all of which succumbed to RVFV challenge by day 3 post-infection. Only 1 of 10 animals receiving DEF201 two weeks prior to challenge survived the infection (Figure 1C); however, the mean time of death was significantly extended out to 5.5 days, 3 days longer than the matched EV-treated group. The protective effect was completely lost when DEF201 was administered 3 weeks prior to RVFV challenge (Figure 1D).

**Figure 2:**
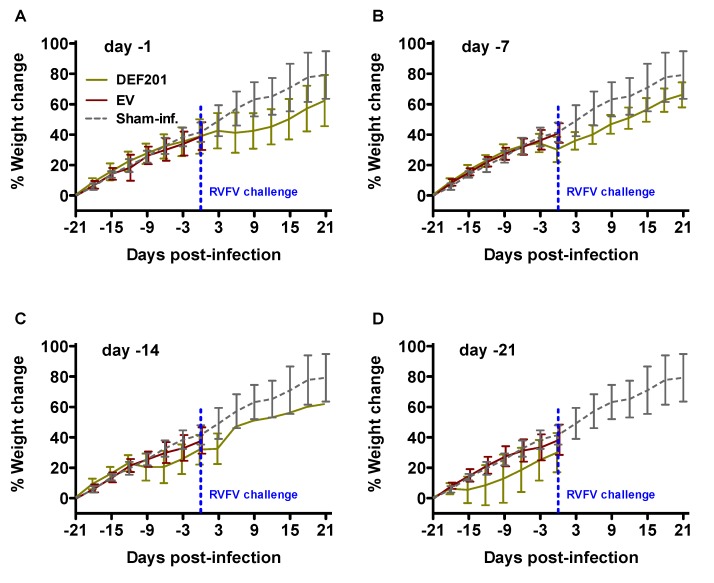
DEF201 treatment results in temporary weight loss. The percent weight change during the course of the DEF201 RVFV pre-exposure prophylaxis experiment presented in Figure 1 is shown. The data for the (**A**) day −1, (**B**) day −7, (**C**) day −14, or (**D**) day −21 treatments are represented as the group mean and standard deviation of the percent change in weight of surviving animals relative to their starting weights on day −21 and measured every 3rd day. Sham-infected normal controls are included for comparison.

Animal weights were also determined during the pre-exposure efficacy study as a general measure of health. The percent weight change of hamsters relative to their starting weights on day −21 relative to RVFV infection are shown in Figure 2. Evidence of weight loss in DEF201 groups in the range of 5%–20% was observed starting several days after each treatment and is consistent with previously reported data indicative of a temporary reduction in food and water consumption in response to the *in situ* production of consensus IFN [10]. The surviving animals all recovered and gained weight at a similar rate to the sham-infected normal controls (Figure 2A–C).

**Figure 3:**
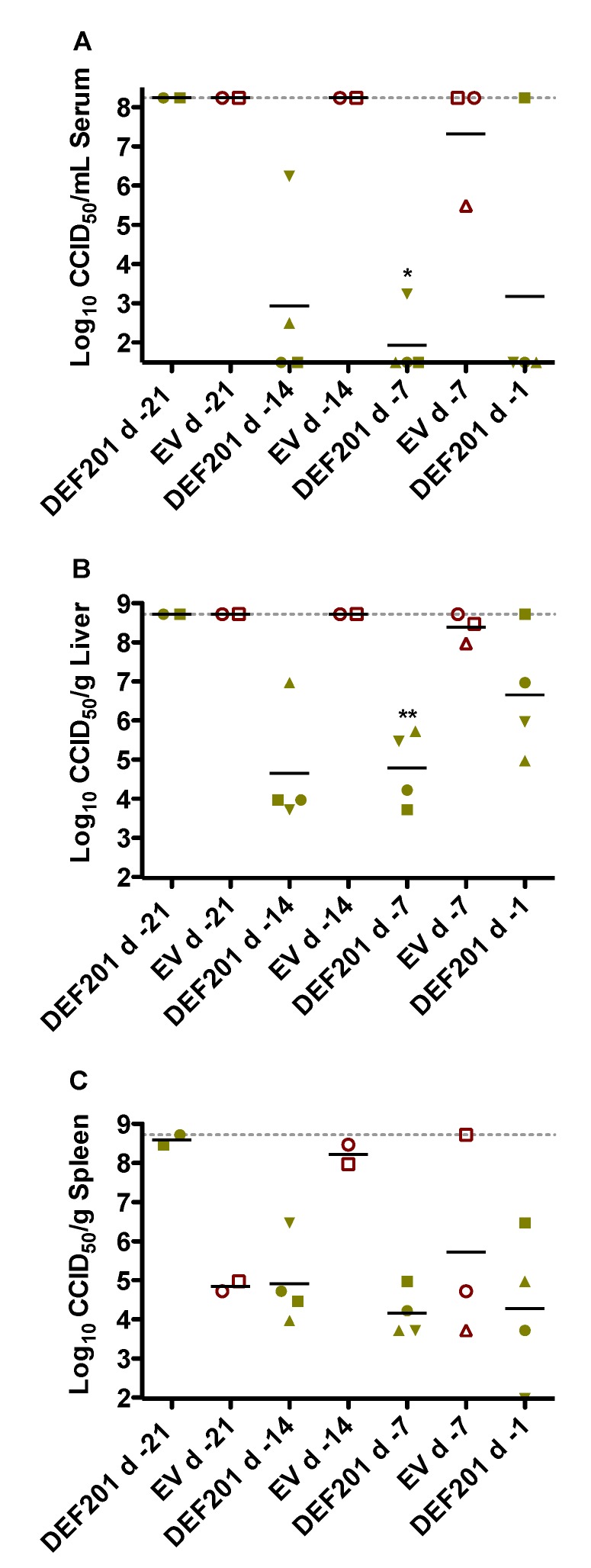
Reduced viral titers associated with protective effect of DEF201. Subgroups of hamsters (n = 4/group) treated and infected in parallel to the animals in the observation groups described in Figure 1 were sacrificed on day 2 post-infection for analysis of (**A**) serum, (**B**) liver, and (**C**) spleen virus titers. Unique symbols in each treatment group represent values for the same animal across all parameters. Several groups have less than 4 data points due to death prior to time of sacrifice. Two animals in the DEF201 d −21 group and nine animals in the EV groups (including all of the d −1 group) succumbed prior to day 2. The gray hashed lines represent the saturation level indicating that actual titers are equal to or greater than the plotted concentration. *p < 0.05, **p < 0.01 compared to respective EV controls.

The antiviral effect of DEF201 pre-exposure treatment on reducing RVFV titers was evaluated on day 2 in a pre-designated subset of hamsters from each experimental group. Despite only very limited protection from mortality in the 14 day DEF201 pre-treatment group, the substantial reduction in serum and tissue viral burden was comparable to that observed with the 1 and 7 day pre-treatments (Figure 3). The two remaining animals in the 21 day pre-treatment group had maximal RVFV titers. Because many of the animals in the EV groups succumbed prior to the time of sacrifice (Figure 3), it was not possible to fully appreciate the impact of DEF201 on viral titers since the sickest animals were not included in the analysis. Notably, the serum and liver viral titers for 1 of the 4 animals in the d −1 DEF201 treatment group were higher than expected (Figure 3A,B). This was unexpected since all the DEF201 d −1 animals observed for morbidity and mortality survived RVFV challenge without any noticeable clinical disease. Because the tissues analyzed were not perfused, the liver and spleen viral titers shown may reflect virus present in the small amount of residual blood remaining in the samples collected.

**Figure 4:**
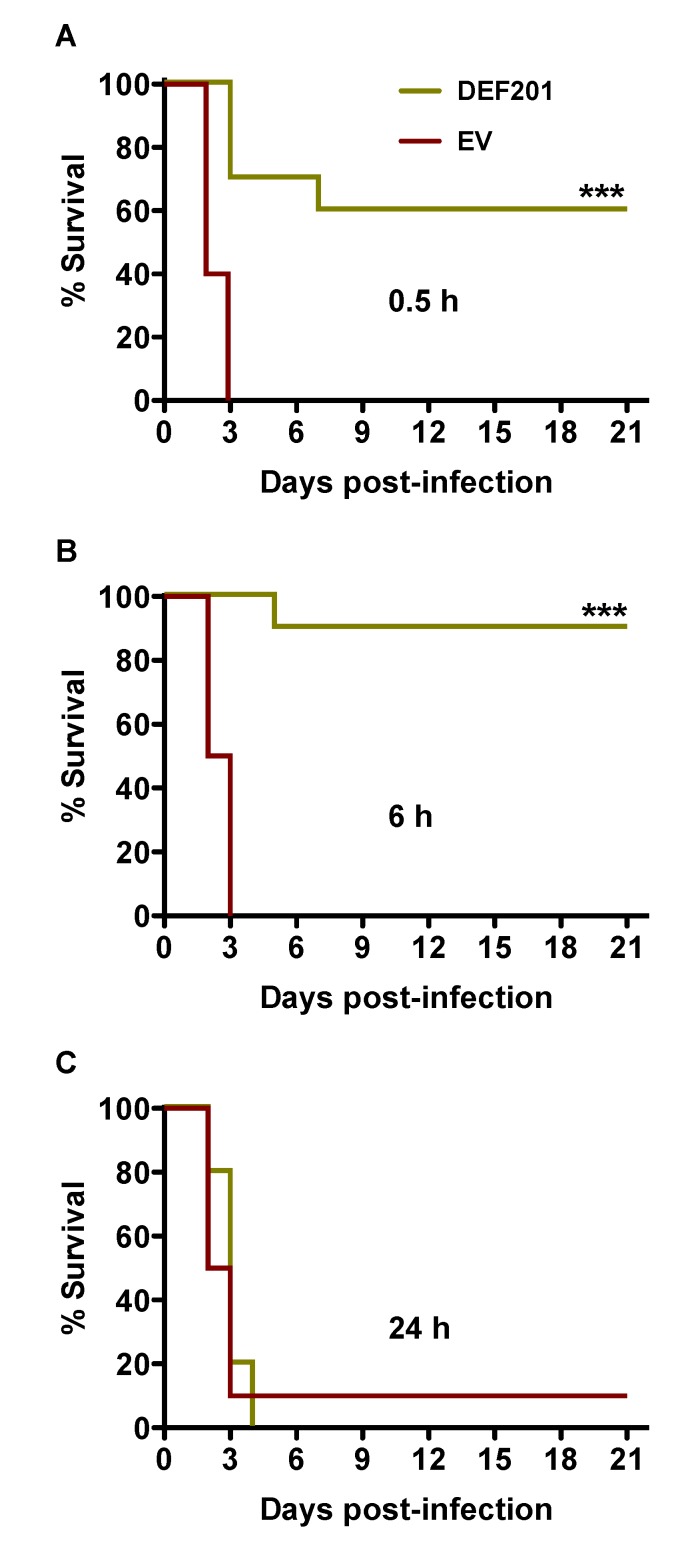
Post-exposure treatment with DEF201 protects hamsters from lethal s.c. RVFV infection. Hamsters (n = 10 per group) were treated with 10^8^ PFU of DEF201 or EV at (**A**) 0.5 h, (**B**) 6 h, or (**C**) 24 h post-infection with 30 PFU of RVFV. Kaplan-Meier survival curves are shown. ***p < 0.001 compared to respective EV controls.

A second experiment was conducted to investigate the potential use of DEF201 as a post RVFV exposure intervention. The treatment times of 0.5, 6 and 24 h post-challenge were selected based on uniform lethality in approximately 2–3 days following s.c. injection with a 30 PFU inoculum. As shown in Figure 1A and B, treatment with a single 10^8^ PFU dose of DEF201 at 0.5 and 6 h post‑infection provided 60% and 90% protection from mortality from lethal RVFV challenge. It is possible that the stress associated with the 0.1 mL i.n. instillation treatment 30 min after RVFV infection had a negative impact on the survival of the DEF201 0.5 h post-infection group. A single hamster that received the EV at 24 h survived the study, while all of the animals receiving DEF201 24 h post-challenge succumbed to the infection by day 4 (Figure 4C).

**Figure 5:**
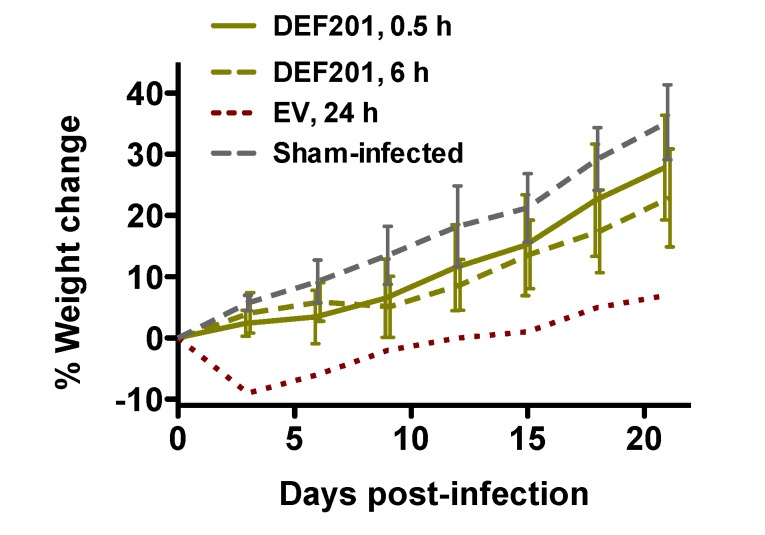
Surviving hamsters recover from initial weight loss due to DEF201 treatment. The percent weight change during the course of the DEF201 RVFV post-exposure treatment experiment presented in Figure 4 is shown. The data are represented as the group mean and standard deviation of the percent change in weight of surviving animals relative to their starting weights on day 0 and measured every 3rd day. Sham-infected normal controls are included for comparison.

Animal weights were also measured during the post RVFV exposure DEF201 efficacy study. The 0.5 and 6 h DEF201 groups showed a slight decrease (5%–10%) in weight gain during the first 6–9 days, and thereafter gained weight at a similar rate to the sham-infected control group (Figure 5). Evidence of weight loss and subsequent recovery in the only surviving animal in the EV group treated at 24 h post-infection indicates that this animal was ill and then recovered from the infection. Due to death prior to weight determinations on day 3, these data are limited to the 0.5 and 6 h DEF201 groups, the 24 h EV group, and the sham-infected controls.

**Figure 6:**
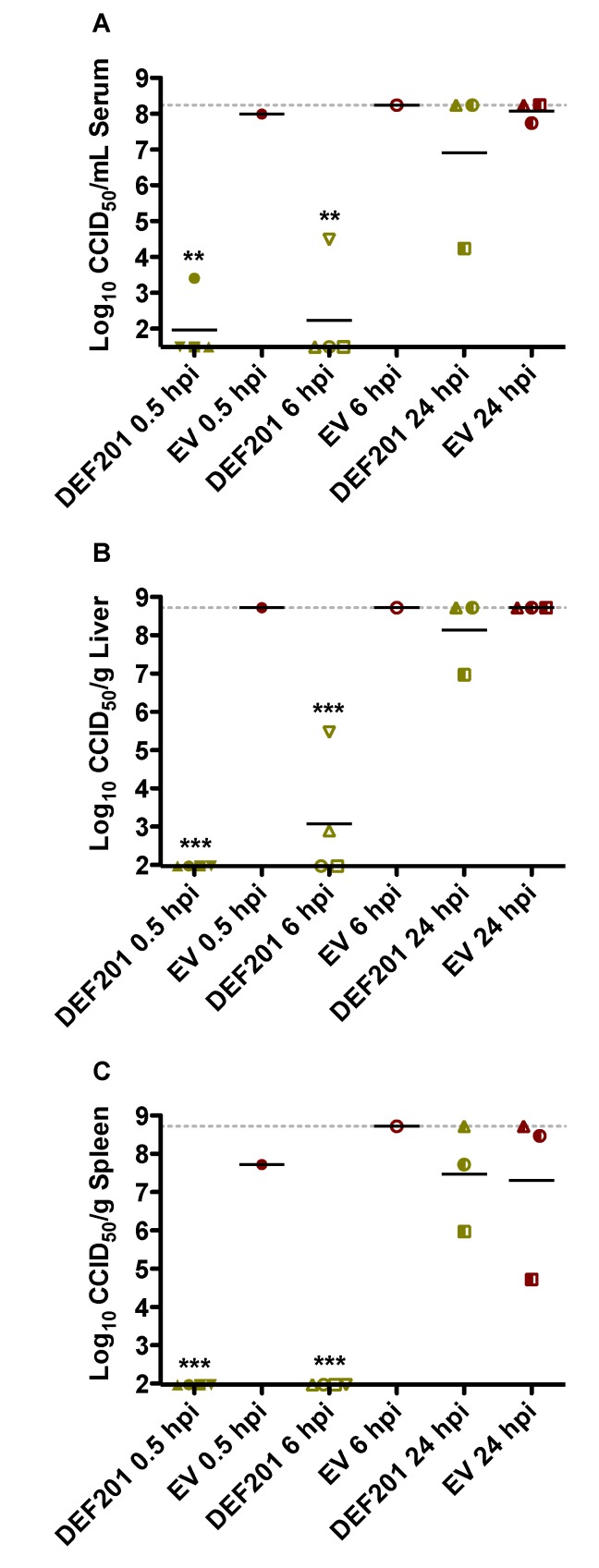
Post-exposure intervention with DEF201 within 6 h of RVFV challenge effectively reduces serum and tissue viral titers. Subgroups of hamsters (n = 4/group) treated and infected in parallel to the animals in the observation groups described in Figure 4 were sacrificed on day 2 post-infection for analysis of (**A**) serum, (**B**) liver, and (**C**) spleen virus titers. Several groups have less than 4 data points due to death prior to time of sacrifice. The gray hashed lines represent the saturation level indicating that actual titers are equal to or greater than the plotted concentration. *p < 0.05, **p < 0.01, ***p < 0.001 compared to the 24 hpi EV control.

As shown in Figure 6, the virus titer data from the post RVFV exposure DEF201 treatment study was consistent with the survival findings (Figure 4). Serum and tissue viral titers assessed on day 2 of infection were dramatically reduced or absent in animals treated within 6 h of RVFV infection, as compared to the EV control groups (Figure 6). Maximal viral loads were observed in most of the available samples from animals in the EV groups. Notably, however, many of the EV-treated hamsters succumbed prior to the time of sacrifice.

## 3. Discussion

Significant advances have been made in the science of predicting RVF epizootics in endemic regions of sub-Saharan Africa several months before the transmission of the virus and the ensuing wave of disease [18,19,20]. This advanced notice could serve to alert public health officials of increased risk in a given area for early implementation of vigorous mosquito vector control measures and prophylactic strategies to limit the disease burden. At present there are no approved antiviral agents available for pre- and post-exposure prophylaxis, which may be useful in limiting RVF disease in high-risk populations such as veterinarians, abattoir workers, herdsmen, and any individuals that work with susceptible ungulate species. In addition, safe and broadly active post-exposure prophylaxis countermeasures would also be of great value to laboratory personnel that study RVF and other severe viral diseases.

In the present study, we show that i.n. treatment with DEF201 one week prior to s.c. challenge of hamsters with a uniformly lethal dose of RVFV was highly efficacious. Greatly reduced but significant protection (*p* < 0.01 by log-rank analysis) was observed with DEF201 treatment administered 2 weeks prior to challenge, suggesting that animals may have been fully protected by treatment initiated several days beyond day 7. To this end, we have previously demonstrated with the 10^8^ PFU dose of DEF201 that systemic levels of cIFN produced from the vector peaks between 4 and 7 days after treatment, and although on the decline, substantial amounts are still detectable after 8 days [10]. Consistent with these data, virus titers reductions were similar in hamsters treated with DEF201 at the 1, 7 or 14 days prior to infection, despite the fact that most of the animals in the 14 day pretreatment group did not survive the challenge. Because of the delay in time of death in the 14 day pretreatment group, it is likely that increased titers would have been resolved at a later sampling time. As a post-exposure intervention, DEF201 was highly efficacious when administered up to 6 h after RVFV challenge. The reduced efficacy observed at the 0.5 h administration time may have been due to the added stress associated with the i.n. treatment shortly after the infection.

DEF201 has been shown to confer complete protection in hamsters challenged with the related Punta Toro phlebovirus when dosed 3 weeks prior to challenge, and 40% survival is observed when treatment is given 4 weeks prior to infection [10]. The difference in the DEF201 prophylactic window between pretreatment of RVFV and PTV infection in hamsters is likely attributed to the reduced susceptibility and less severe disease in the latter. Most of the hamsters receiving the EV control virus devoid of the consensus IFN gene encoded in DEF201 succumbed within two days of the RVFV challenge, with the remaining animals expiring by the third day. In contrast, PTV infection is often sublethal with a s.c. challenge dose of 50 PFU and results in a more protracted disease [10]. Thus, despite the encouraging results, it is difficult to translate the findings in hamsters to the human condition because of the peracute nature of the disease and exquisite sensitivity of the animals to the ZH501 strain of RVFV [21,22,23,24]. In fatal cases of RVF, humans can succumb within 3–6 days of the onset of clinical illness, but death can also occur in some cases more than two weeks after the initial signs of disease [3]. Fortunately, most infections result in only mild to moderate illness with estimated overall case fatality rates in the 0.5%–2% range [25]. Based on the virus titer data in the pre-exposure prophylaxis setting (Figure 3), it is likely that humans prophylactically given DEF201 would become ill due to some level of viral replication, but most would control the infection through the effects of cIFN sufficiently enough to prevent severe forms of RVF including hemorrhagic disease, acute hepatitis, late-onset encephalitis and retinitis that can lead to varying degrees of blindness. Naturally, the RVFV hamster infection model does not reproduce the neurologic or ocular disease seen in some human cases; however, we recently reported the development of late-onset encephalitis following ribavirin treatment of RVFV-infected hamsters [24]. RVFV infection in ACI rats and common marmosets has been shown to produce encephalitis and, therefore, may be good models for evaluating DEF201 against the development of neurologic disease [26,27]. 

We did not evaluate DEF201 against i.n. RVFV challenge, but based on slower disease progression and a higher LD_50_ [28], we would predict a broader post-exposure prophylaxis window. We also did not investigate whether protective immunity to re-challenge with RVFV was induced in surviving animals. However, in previous work in the hamster PTV and Pichinde arenavirus infection models, there was a direct correlation between survival to a second challenge and moderate levels of viral replication [7,10]. Based on these data, it is likely that in cases where the effects of DEF201 did not fully prevent RVFV replication (Figure 3), the adaptive immune response would confer protective immunity against repeat infection. Thus, we would expect that the surviving animals from the pre-exposure study would have been much more refractory to secondary challenge compared to hamsters from the 0.5 and 6 h post-exposure treatment groups where the effect of DEF201 was sterilizing in many cases (Figure 6).

Administration of adenovirus type 5 vectors via the i.n. route has been demonstrated to effectively bypass circulating pre-existing immunity to the vector, allowing the proteins expressed to induce protective immunity [29,30]. This allows for the usage of these effective and clinically-experienced viruses as gene delivery vectors for a wide range of applications, including pre- and post-exposure prophylaxis against pathogenic viruses. The data presented supports further investigation into the potential use of DEF201 to prevent or limit RVF, especially in nonhuman primate models which would be more predictive of the prophylactic capacity of DEF201. In addition to public health implications of the disease in endemic regions of Africa and neighboring countries, the heightened risk of introduction into the United States, Europe, and other areas of the world underscore the need to develop simple, cost-effective, and broadly active countermeasures, such as DEF201, to combat accidental or intentional release of RVFV into naïve regions where reservoir species and hosts susceptible to fulminant disease are most vulnerable [31].

## 4. Experimental Section

### 4.1. Animals

Female golden Syrian hamsters were obtained from Charles River (Willimantic, CT, USA). The hamsters were quarantined for 7 day prior to each experiment and maintained on Harlan Lab Block and tap water *ad libitum*. All animal procedures used in this study complied with guidelines set by the USDA and Utah State University Animal Care and Use Committee.

### 4.2. Viruses

The infectious clone of RVFV, strain ZH501, was obtained from Dr. Stuart Nichol (CDC, Atlanta, GA, USA). The virus stock (1 passage in BSRT7 cells, 3 passages in Vero E6 cells) used was titrated to have a concentration of 1.1 × 10^8^ PFU/mL and was derived from a clarified cell culture lysate preparation. The virus inoculum was prepared by dilution in sterile medium and inoculated by s.c. injection of 0.1 mL (ventral, right side of the abdomen).

The DEF201 adenovirus and the adenovirus empty vector (EV) control were provided by Defyrus (Toronto, Ontario, Canada) at a concentration of 6 × 10^9^ PFU/mL and 2 × 10^11^ PFU/mL, respectively. Both recombinant adenoviruses were prepared in sterile saline for i.n. instillation in 0.1 mL volumes. 

### 4.3. Experimental Design

*Experiment 1*. Hamsters were weighed on day −21 relative to the day of infection and grouped so that the average weight per group (n = 14) across the entire experiment varied by less than 5 grams. Animals in each group (70–85 g range) were treated i.n. once with 10^8^ PFU of DEF201 or EV at either −21, −14, −7, or −1 day prior to s.c. challenge with 30 PFU (10 × LD_50_) of RVFV. Infection by the s.c. route models the most common mode of transmission by mosquitoes. Four animals from each treatment group were designated for sacrifice on day 2 of infection for analysis of serum and tissue viral titers. The remaining animals were observed for 21 days post-infection for morbidity and mortality. Sham-infected animals were included as normal controls for morbidity and mortality (n = 4) and to define detection limits for virus titer assays (n = 2). 

*Experiment 2*. Hamsters were weighed on the day of infection and grouped so that the average weight per group (n = 14 for each treatment and placebo group) across the entire experiment varied by less than 6 grams. Animals in each group (88–121 g) were challenged by s.c. injection with 30 PFU of RVFV and subsequently treated by a single i.n. instillation of 10^8^ PFU of DEF201 or EV at either 0.5, 6, or 24 h post-challenge. Four animals from each treatment group were designated for sacrificed on day 2 of infection for analysis of serum and tissue viral titers and the remaining hamsters were observed 21 day for morbidity and mortality. Sham-infected normal animals were included as controls for morbidity and mortality (n = 3) and virus titer assays (n = 3).

### 4.4. Serum, Liver, and Spleen Virus Titers

Virus titers were assayed using an infectious cell culture assay as previously described [32]. Briefly, a specific volume of serum or liver or spleen homogenate was serially diluted and added to triplicate wells of Vero (African green monkey kidney) cell monolayers in 96-well microtiter plates. The viral cytopathic effect was determined 7 days after plating and the 50% endpoints were calculated as described [33]. The upper and lower limits of detection for serum titer were 8.24 and 1.49 log_10_ 50% cell culture infectious doses (CCID_50_)/mL, respectively. The upper and lower limits of detection for tissues were 8.72 and 1.97 log_10_ CCID_50_/g of tissue, respectively. In samples with saturated or undetectable levels of virus, a value representative of the respective limit of detection was assigned for statistical analysis.

### 4.5. Statistical Analysis

The Mantel-Cox log-rank test was used for analysis of Kaplan-Meier survival curves. A one-way analysis of variance (ANOVA) with a Newman-Keuls posttest was performed to compare differences in virus titers. All statistical evaluations were done using Prism software, version 5.0d (GraphPad Software, La Jolla, CA, USA) [34].

## 5. Conclusions

Our findings indicate that DEF201, as a single dose i.n. treatment, is highly effective as a pre- and post-exposure prophylactic measure against acute RVFV infection in hamsters. Further studies using nonhuman primate models of RVF will evaluate DEF201’s utility for use in the event of an accidental laboratory exposure or during a RVF epizootic outbreak.
